# Mental Health and Well-Being of University Students: A Bibliometric Mapping of the Literature

**DOI:** 10.3389/fpsyg.2020.01226

**Published:** 2020-06-09

**Authors:** Daniel Hernández-Torrano, Laura Ibrayeva, Jason Sparks, Natalya Lim, Alessandra Clementi, Ainur Almukhambetova, Yerden Nurtayev, Ainur Muratkyzy

**Affiliations:** ^1^Graduate School of Education, Nazarbayev University, Nur-Sultan, Kazakhstan; ^2^Nazarbayev University School of Medicine, Nur-Sultan, Kazakhstan; ^3^Psychological Counseling Center, Nazarbayev University, Nur-Sultan, Kazakhstan

**Keywords:** mental health, mental illness, well-being, psychological distress, university students, higher education, bibliometric review, VOSViewer

## Abstract

The purpose of this study is to map the literature on mental health and well-being of university students using metadata extracted from 5,561 journal articles indexed in the Web of Science database for the period 1975–2020. More specifically, this study uses bibliometric procedures to describe and visually represent the available literature on mental health and well-being in university students in terms of the growth trajectory, productivity, social structure, intellectual structure, and conceptual structure of the field over 45 years. Key findings of the study are that research on mental health and well-being in university students: (a) has experienced a steady growth over the last decades, especially since 2010; (b) is disseminated in a wide range of journals, mainly in the fields of psychology, psychiatry, and education research; (c) is published by scholars with diverse geographical background, although more than half of the publications are produced in the United States; (d) lies on a fragmented research community composed by multiple research groups with little interactions between them; (e) is relatively interdisciplinary and emerges from the convergence of research conducted in the behavioral and biomedical sciences; (f) tends to emphasize pathogenic approaches to mental health (i.e., mental illness); and (g) has mainly addressed seven research topics over the last 45 years: positive mental health, mental disorders, substance abuse, counseling, stigma, stress, and mental health measurement. The findings are discussed, and the implications for the future development of the field are highlighted.

## Introduction

The entrance to the university marks a period of transition for young people. Through this transition, students face new challenges, such as making independent decisions about their lives and studies, adjusting to the academic demands of an ill-structured learning environment, and interacting with a diverse range of new people. In addition, many students must, often for the first time, leave their homes and distance themselves from their support networks (Cleary et al., 2011). These challenges can affect the mental health and well-being of higher education students. Indeed, there is evidence that a strain on mental health is placed on students once they start at the university, and although it decreases throughout their studies (Macaskill, 2013; Mey and Yin, 2015), it does not return to pre-university levels (Cooke et al., 2006; Bewick et al., 2010). Also, the probabilities of experiencing common psychological problems, such as depression, anxiety, and stress, increase throughout adolescence and reach a peak in early adulthood around age 25 (Kessler et al., 2007) which makes university students a particularly vulnerable population.

The interest in mental health and well-being in university students has grown exponentially in the last decades. This is likely due to three interrelated challenges. First, although university students report levels of mental health similar to their non-university counterparts (Blanco et al., 2008), recent studies suggest an increase and severity of mental problems and help-seeking behaviors in university students around the world in the last decade (Wong et al., 2006; Hunt and Eisenberg, 2010; Verger et al., 2010; Auerbach et al., 2018; Lipson et al., 2019). Some researchers refer to these trends as an emerging “mental health crisis” in higher education (Kadison and DiGeronimo, 2004; Evans et al., 2018). Second, psychological distress in early adulthood is associated with adverse short-term outcomes, such as poor college attendance, performance, engagement, and completion (e.g., King et al., 2006; Antaramian, 2015), and others in the long term, such as dysfunctional relationship (Kerr and Capaldi, 2011), recurrent mental health problems, university dropout, lower rates of employment, and reduced personal income (Fergusson et al., 2007). Third, there is a widespread agreement that higher education institutions offer unique opportunities to promote the mental health and well-being of young adults as they provide a single integrated setting that encompasses academic, professional, and social activities, along with health services and other support services (Eisenberg et al., 2009; Hunt and Eisenberg, 2010). However, the majority of university students experiencing mental health problems and low levels of well-being are not receiving treatment (Blanco et al., 2008; Eisenberg et al., 2011; Lipson et al., 2019) and, while universities continue to expand, there is a growing concern that the services available to provide support to students are not developing at an equivalent rate (Davy et al., 2012).

In response to the increasing volume of research on the mental health and well-being of university students, there have been several attempts to synthesize the accumulating knowledge in the field and to provide an illustration of the theoretical core and structure of the field using traditional content analysis of the literature (e.g., Kessler et al., 2007; Gulliver et al., 2010; Hunt and Eisenberg, 2010; Sharp and Theiler, 2018). This study aims to extend the understanding of mental health in university students by providing a bird’s eye view of the research conducted in this field in recent decades using a bibliometric approach. Bibliometric overviews provide an objective and systematic approach to discover knowledge flows and patterns in the structure of a field (Van Raan, 2014) reveal its scientific roots, identify emerging thematic areas and gaps in the literature (Skute et al., 2019) and, ultimately, contribute to moving the field forward. Accordingly, this study employs several bibliometric indicators to explore the evolution of the field based on publication and citation trends, key actors and venues contributing to the advancement of research on mental health and well-being of university students, and the structure of the field in terms of patterns of scientific collaborations, disciplines underlying the foundations of the field, and recurrent research themes explored in the literature. This is important because, despite significant advances in the field, research on mental health and well-being remains a diverse and fragmented body of knowledge (Pellmar and Eisenberg, 2000; Bailey, 2012; Wittchen et al., 2014a). Indeed, mental health and well-being are nebulous concepts and their history and development are quite intricate, with a multitude of perspectives and contributions emerging from various disciplines and contexts (see section “Conceptualization of Mental Health, Mental Illness, and Well-Being: An Overview”). Therefore, mapping research on mental health and well-being in university students is essential to identify contributions and challenges to the development of the field, to help guide policy, research, and practice toward areas, domains, populations, and contexts that should be further explored, and to provide better care of students at higher education institutions (Naveed et al., 2017).

## Conceptualization of Mental Health, Mental Illness, and Well-Being: An Overview

This section provides an overview of the different perspectives adopted in the literature to conceptualize mental health, well-being, and other relevant constructs in order to identify the glossary of key terms that will be used in the search strategy to create a comprehensive corpus of documents on mental health and well-being in university students for this bibliometric review.

### Perspectives on Mental Health and Mental Illness

There is no general agreement on the definition of mental health. For a long time, the term mental health has been used as a euphemism for mental illness (Manwell et al., 2015). However, mental health and mental illness are regarded as distinct constructs nowadays and two main perspectives differentiating between mental health and illness are available in the literature. The continuum approach considers that mental health and mental illness are the two opposite poles of a continuum. Thus, there are various degrees of health and illness between these poles, with most of us falling somewhere in between. The categorical approach, on the other hand, represents mental health and illness as a dichotomy. People who manifest mental illness symptoms would belong to that category and labeled correspondingly, while those absent of these symptoms can be considered as mentally healthy (Scheid and Brown, 2010).

### Disciplinary Approaches to the Conceptualization of Mental Health/Illness

Conceptualizations of mental health/illness are largely dependent on the theoretical and paradigmatic foundations of the disciplines from which they emerge. In this context, the field has progressively evolved through the accumulation of knowledge generated in a diverse range of disciplines in the biomedical, behavioral, and social sciences. Biomedical disciplines are grounded in the medical paradigm focused on disease and (ab)normality and often emphasize dichotomous conceptions of mental health/illness (Scheid and Brown, 2010). Research on mental health and well-being in this domain has been traditionally conducted from a psychiatric perspective, which aims to understand the dysfunctionality in the brain that leads to psychiatric symptoms and to also offer a pharmacological treatment to correct neuronal dysfunctions. Consequently, psychiatrists have historically considered mental health as a disease of the brain (e.g., depression), similar to any other physical disease, caused by genetic, biological, or neurological factors (Schwartz and Corcoran, 2010). While the prevalence of psychiatric approaches to mental health is currently incontestable, the development of other biomedical disciplines has tremendously contributed to the progression of the field in recent decades. For example, Insel and Wang (2010) argue that insights gained from genetics and neuroscience contribute to the reconceptualization of “the disorders of the mind as disorders of the brain and thereby transform the practice of psychiatry.” (1979). In addition to that, other disciplines such as behavioral medicine have made important contributions to the field, although it has recently argued that mental health and behavioral medicine should be as two separate fields (Dekker et al., 2017).

Within the behavioral sciences, the study of mental health focuses on the distinct psychological processes and mechanisms that prompt thoughts, feelings, and behaviors (Peterson, 2010). Clinical psychology has the longest tradition in the psychological study of mental health and tends to focus on the assessment and treatment of mental illness and disorders that can alleviate psychological distress or promote positive states of being (Haslam and Lusher, 2011). However, significant contributions to the field have also emerged from other branches of psychology less focused on psychopathology, including personality and social psychology, psychoanalysis, humanistic psychology, and cognitive psychology (Peterson, 2010). Despite the diversity of theories, principles, and methodological approaches to understanding mental health within the behavioral sciences, these disciplines acknowledge that mental health have a biological basis and reside in the social context, and tend to prioritize continuum approaches to mental health (Scheid and Brown, 2010).

Perspectives from the social sciences complement the biomedical and behavioral approaches by considering the influence of social and cultural environments in mental health/illness (Horwitz, 2010). For example, sociologists are interested in how social circumstances (e.g., level of support available) affect levels of mental health/illness and how social structures shape the understanding and response to mental health issues [see Compton and Shim (2015) for an overview of the social determinants of mental health]. Similarly, medical anthropologists attend to the mental health beliefs and practices that form the cultural repertory within and across populations (Foster, 1975). Beyond sociology and anthropology, social researchers in the fields of business and economics, family and ethnic studies, and educational research have also played a key role in advancing research on mental health in different directions.

### The Importance of the Context in Mental Health

Certainly, most notions of mental health/illness in the literature derive from prevailing psychiatric and psychological traditions developed in Western countries (Gopalkrishnan, 2018). However, cultural values and traditions do shape how mental health and mental illness are conceptualized across contexts (Vaillant, 2012). In this regard, Eshun and Gurung (2009) pointed out that “culture influences how individuals manifest symptoms, communicate their symptoms, cope with psychological challenges, and their willingness to seek treatment.” (4). Fernando (2019) argued that issues related to the ‘mind’ developed and are often interpreted very differently in non-Western and Low- and Middle-Income Countries (LMICs). For example, cultures explain the manifestation of certain feelings and behaviors based on a range of motives including biological, psychological, social, religious, spiritual, supernatural, and cosmic. Failure to acknowledge alternative non-Western approaches to mental health and mental illness has resulted in imbalances of knowledge exchange and the permeation of dominating Western narratives into LMICs (i.e., so-called medical imperialism) (Timimi, 2010; Summerfield, 2013). To address this issue, scholars have advocated for a greater willingness to embrace pluralism in the conceptualization of mental health and illness, which might help people to engage with particular forms of support that they deem to be appropriate for them, and to explore how knowledge and practices developed in LMICs can benefit those living in higher-income countries (i.e., knowledge “counterflow”) (see White et al., 2014).

### Prioritizing Positive Mental Health: The Science of Well-Being

Despite the diversity of disciplinary and contextual approaches to mental health, current definitions of mental health have two things in common. First, mental health is considered from a *biopsychosocial* point of view that incorporates biological, psychological, and social factors. Second, mental health implies something beyond the absence of mental illness (e.g., Bhugra et al., 2013; Galderisi et al., 2015). An example is the definition by the World Health Organization which refers to mental health as “a state of well-being in which every individual realizes his or her own potential, can cope with the normal stresses of life, can work productively and fruitfully, and is able to make a contribution to her or his community” (World Health Organization, 2004). This definition contributed to substantial progress in research and practice in the field as it expanded the notion of mental health beyond the absence of mental illness and integrated the presence of positive features (Galderisi et al., 2015).

Research on positive mental health is relatively new but has grown rapidly in the last decades fueled by advocates of positive medicine and psychology, who have argued for a change of paradigm from medical and psychopathological-oriented models of mental health that focus on disorders and illness toward more strength-based approaches, which pay more attention to what is right about people and positive attributes and assets (Kobau et al., 2011). In this regard, the term mental well-being has been progressively incorporated into the study of mental health to account for the positive aspects of mental health beyond the absence of negative factors. While there is not a universally accepted definition of well-being, two perspectives have dominated the discourses on well-being in the literature: subjective well-being (SBW) and psychological well-being (PWB). SWB is based on hedonic perspectives of pleasure and represents “people’s beliefs and feelings that they are living a desirable and rewarding life” (Diener, 2012). SBW is strongly linked with the idea of happiness and is typically understood as the personal experience of high levels of positive affect, low levels of negative affect, and high satisfaction with one’s life (Deci and Ryan, 2008). PWB is grounded in Aristotelian ideas about eudaimonia, i.e., self-realization, with the ultimate aim in life being to strive to realize one’s true potential (Ryff and Singer, 2008). PWB has been broadly defined as a state of positive psychological functioning and encompasses six dimensions: purpose in life (i.e., the extent to which respondents felt their lives had meaning, purpose, and direction); autonomy (i.e., whether they viewed themselves as living in accord with their own convictions); personal growth (i.e., the extent to which they were making use of their personal talents and potential); environmental mastery (i.e., how well they were managing their life situations); positive relationships (i.e., the depth of connection they had in ties with significant others); and self-acceptance (i.e., the knowledge and acceptance they had of themselves, including awareness of personal limitations) (Ryff, 1989).

### Integrating Mental Health, Mental Illness, and Well-Being

The contribution of positive mental health frameworks to the advancement of the field has been undeniable. However, definitions that overemphasize positive emotions and productive functioning as key indicators of mental health have been recently challenged because of the potential they have to discriminate against individuals and groups that, for example, might not be able to work productively or function within the environment because of individual physical characteristics or contextual constraints (Galderisi et al., 2015). To address these issues, Keyes has successfully integrated the notions of mental illness, mental health, well-being, and other related terms in the literature into a conceptual framework that allows for a more comprehensive understanding of mental health (Keyes, 2005, 2007; Keyes and Michalec, 2010). The model argues that neither pathogenic approaches focusing on the negative (e.g., mental illness) nor salutogenic approaches focusing on the positive (e.g., well-being) can alone accurately describe the mental health of a person (Keyes and Michalec, 2010). Instead, the model proposes that mental illness and well-being represent two correlated but differentiated latent continua in defining mental health. More specifically, mental illness and well-being lie on two separate spectra, the first going from absent to present mental illness and the second running from low to high well-being (Slade, 2010). The absence of mental illness, therefore, does not necessarily imply high levels of well-being. Correspondingly, low levels of well-being do not always indicate the presence of mental illness. Further, in this model, mental health is defined as not only the absence of mental illness, not the mere presence of high well-being. Complete mental health (i.e., flourishing) is a result of experiencing low mental illness and high levels of well-being. Incomplete mental health (i.e., languishing), on the other hand, refers to the absence of mental illness symptoms and low reported levels of well-being. Two other conditions are possible within this framework. Incomplete mental illness (i.e., struggling) refers to high levels of well-being accompanied by high mental illness symptoms. Lastly, complete mental illness (i.e., floundering) accounts for low levels of well-being and high mental illness symptoms (Keyes and Lopez, 2002).

## The Present Study

In light of the complexity of the constructs of mental health and well-being and the multiple theoretical, disciplinary, and contextual approaches to their conceptualization, this study seeks to map out the terrain of international research and scholarship on mental health and university students for the period 1975–2020. More specifically, this study aims to provide new insights into the development and current state of mental health research in university students by mapping and visually representing the literature on mental health and well-being of university students over the last 45 years in terms of the growth trajectory, productivity, and social, intellectual, and conceptual structure of the field. First, the study describes the development of research mental health and well-being in university students examining the trends in publication and citation data between 1975 and 2020 (i.e., growth trajectory). Second, the study identifies the core journals and the research areas contributing most to the development of the field, as well as the key authors and countries leading the generation and dissemination of research on mental health and well-being in university populations (i.e., productivity). Third, the study outlines the networks of scientific collaboration between authors, and countries (i.e., social structure). Fourth, the scientific disciplines underlying the intellectual foundations of research on mental health and well-being in university settings (i.e., intellectual structure) are uncovered. Fifth, the study elucidates the topical foci (i.e., conceptual structure) of the research on the mental health and well-being of university students over the last 45 years.

## Materials and Methods

A bibliometric approach was used in this study to map the literature on mental health and well-being in university students over the last 45 years using metadata extracted from four indexes of the Web of Science (WoS): The Science Citation Index-Expanded (SCI-Expanded); the Social Sciences Citation Index (SSCI); the Arts & Humanities Citation Index (A&HCI); and the Emerging Sources Citation Index (ESCI). Several reasons justified the selection of the WoS database in this study. First, the WoS remains as the standard and most widely used for bibliometric analysis (Meho and Yang, 2007). Second, the WoS is a multidisciplinary database and includes publications on mental health and well-being emerging from distinctive research areas and disciplines published in more than 20,000 journals (McVeigh, 2009). Using specialized databases such as PubMed would introduce biases into the search strategy favoring biomedical research disciplines. Still, it is important to note that interdisciplinary databases such as WoS and Scopus discriminate against publications in the Social Sciences and Humanities and publications in languages other than the English language (Mongeon and Paul-Hus, 2016), so the picture provided by WoS is still imperfect. Third, while other databases might provide wider coverage, WoS includes publication and citation information from 1900. For example, Scopus has complete citation information only from 1996 (Li et al., 2010). Moreover, Google Scholar provides results of inconsistent accuracy in terms of citations, and citation analyses in PubMed are not available (Falagas et al., 2008). Fourth, WoS has demonstrated better accuracy in its journal classification system compared to Scopus database (Wang and Waltman, 2016).

**FIGURE 1:**
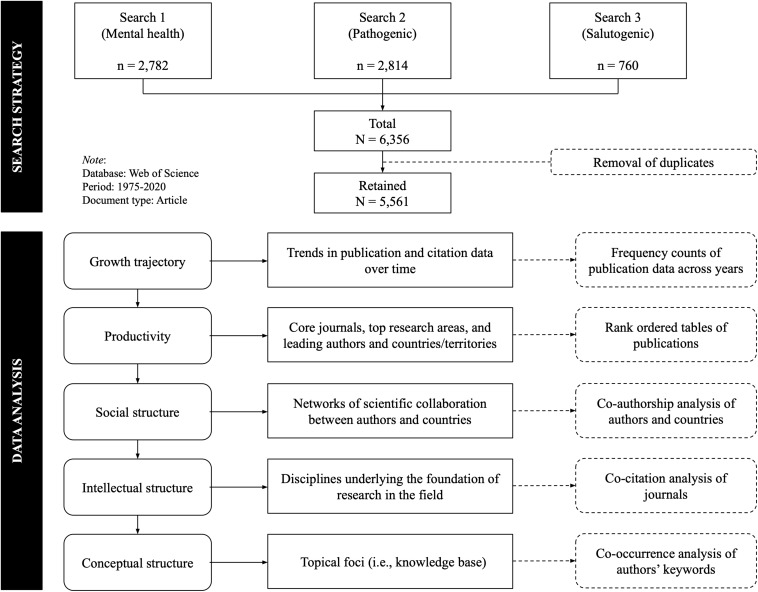
Methodological framework.

The methodological approach used in this study is presented in Figure 1 and further elaborated in the following paragraphs.

### Search Strategy

To create a comprehensive corpus of documents on the mental health and well-being of university students, three parallel searches were performed, which accounted for the multiple approaches and perspectives that have been used in the field, as identified in the Section “Conceptualization of Mental Health, Mental Illness, and Well-Being: An Overview.” All the searches were conducted in the last week of January 2020. The first search aimed at capturing research on *mental health broadly* and included one single keyword in the topic field: [“mental health”]. The second search was implemented to capture research focusing on *pathogenic* approaches to mental health. Key terms used in the literature to refer to the negative side of mental health, as well as the most frequent mental health problems experienced by university students, were introduced in this search in the title field: [“mental illness,” “mental disorder^∗^,” “mental distress,” “psychological distress,” “psychopathology,” “depression,” “anxiety,” “stress,” “suicide,” “eating disorder^∗^,” “substance use”]. In the third search, keywords reflecting *salutogenic* approaches to mental health were input. These included terms related to mental health from a positive mental health perspective (i.e., well-being). These key terms were added in the title field and included the following: [“well-being,” “wellbeing,” “wellness,” “life satisfaction,” “happiness,” “positive affect,” “purpose in life,” “personal growth,” “self-determination”].

To retrieve research relevant only to higher education students, another set of keywords was imputed in all three searches in the title field. These included: [“university,” “college,” “higher education,” “tertiary education,” “post-secondary education,” “postsecondary education,” “undergrad^∗^ student,” “grad^∗^ student,” “master’s student,” “doctoral student,” “Ph.D. student”]. The Boolean operator *OR* was used between keywords in all the three searches to secure a higher number of relevant hits. Also, asterisks were used as wildcards to account for multiple variations in several keywords (e.g., disorder and disorder-s). All searches were limited to journal articles published between 1975 and 2020 (both inclusive). No restrictions on language were implemented in the search.

The search strategy retrieved a total of 6,356 hits (*n*_*search 1*_ = 2782; *n*_*search 2*_ = 2814, *n*_*search 3*_ = 760). After the removal of duplicates, 5,561 research articles were finally selected and retained for the study. For each of the documents obtained in the search, the authors extracted metadata about the title of the paper, the year of publication, the journal, the number of citations, and the authors’ name, organization, and country. Also, the title, the abstract, the author’s keywords, and cited references were retrieved.

### Data Analysis Procedures

The corpus of the literature was then analyzed using descriptive and bibliometric approaches to provide an overall picture of the evolution and current state of the research on mental health and wellbeing in university settings. Frequency counts of the number of publications and citations per year were obtained to describe the growth trajectory of research on the mental health and well-being of university students. Rank ordered tables were produced to describe the productivity of the field in terms of core journals and research areas, as well as leading scholars and countries contributing to the development of the field.

Bibliometric analyses in VOSViewer software were implemented to examine and visually represent the social, intellectual, and conceptual structure of the field. VOSViewer is a freely available computer software for viewing and constructing bibliometric maps^1^. In VOSViewer, the units of analysis are journals, publications, citations, authors, or countries, depending on the focus of the analysis. The units of analysis are represented in the maps as circular nodes. The size of the node accounts for volume (e.g., number of publications in the dataset by an author) and the position represents the similarity with other nodes in the map. Closer nodes are more alike than nodes far apart from each other. The lines connecting nodes represent the relationship between nodes and their thickness indicates the strength of that relationship. Finally, the color of the node denotes the cluster to which each node has been allocated. Nodes are clustered together based on relatedness (Van Eck et al., 2010). The software uses a distance-based approach to constructing the bibliometric maps in three steps (Van Eck and Waltman, 2014). In the first step, the software normalizes the differences between nodes. In the second step, the software builds a two-dimensional map where the distance between the nodes reflects the similarity between these nodes. In the third step, VOSViewer groups closely related nodes into clusters (Van Eck and Waltman, 2014).

A series of co-authorship analyses were performed to examine the social structure of research on mental health and well-being in university students. In these analyses, the units of analysis were authors and countries/territories. Each node in the map represents an author or a country/territory and the lines connecting them reflect the relationship between nodes. Clusters represent networks of scientific collaboration, which might be interpreted as groups of authors or countries frequently publishing together (e.g., research groups in the case of authors).

Co-citation analysis of journals was implemented to explore the intellectual structure of the field. Here, the units of analysis were journals in the dataset and the map reflects co-citation relationships between journals. Two journals are co-cited if there is a third journal citing these two. The more times a pair of journals are cited by other journals, the stronger their co-citation relationship will be. Frequently co-cited journals are assumed to share theoretical and semantical grounds. Therefore, in our study, clusters of frequently co-cited journals can be interpreted as disciplines underlying the foundations of research on mental health and well-being in university students.

Finally, a co-occurrence analysis of keywords was used to uncover the conceptual structure of the field. The units of analysis, in this case, were the authors’ keywords. The more often two keywords appear in the same record, the stronger their co-occurrence relationship. Clusters of co-occurring keywords represent in this study the topical foci (i.e., knowledge base) that have been addressed in the literature in mental health and well-being in university students in the last 45 years.

## Findings and Discussion

### Growth Trajectory: Evolution of Publications and Citations in the Field

**FIGURE 2:**
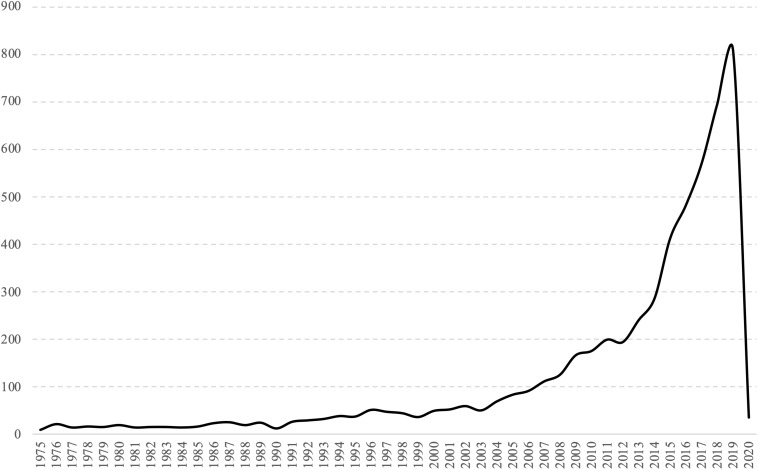
Growth of research on mental health and well-being of university students.

The developmental patterns of a particular field can be well demonstrated by trends in publications and citations. The 5,561 publications in the dataset have been cited 87,096 times, with an average of 15.6 citations per item. Figure 2 shows the growth trajectory of publication data of research on mental health and well-being in university students from 1975 to January 2020. Overall, the trends demonstrate a gradual increase in the scholarly interest in the mental health of university students over the last 45 years that can be organized in three stages: an emergence stage, in which publications rose slowly (1975–2000); a fermentation stage, with a notable increase in publications in the field (2000–2010); and a take-off stage, during which the number of records published per year in the field has almost risen 10 times (2010–2020). The steady increase of publications in the last 15 years coincides with the first calls for attention on the increase and severity of mental problems and help-seeking behaviors of college students (Kadison and DiGeronimo, 2004; Evans et al., 2018), potentially indicating a growing interest in exploring the epidemiology of mental disorders and the role of universities in promoting the mental health and well-being of students. A similar pattern has also been observed in a recent bibliometric study examining global research on mental health both in absolute terms and as a proportion of all papers published in medicine and across disciplines, which certainly reflects an increase in the general interest in the field (Larivière et al., 2013).

### Productivity I: Core Journals and Research Areas

In total, 1,560 journals published the 5,561 records included in the dataset. Table 1 presents the ten core journals in the field. The *Journal of American College* stands out as the main publication venue in the field, accumulating around 5% of the publications in the dataset (*n* = 270). *Psychological Reports* and *Journal of College Student Development* also stand out, publishing 119 and 102 studies, respectively. The *Journal of Counseling Psychology* ranks fourth in the list with 83 records. Despite being an interdisciplinary and relatively young journal, *Plos One* appears in the top five journal publishing research on mental health and well-being in university students.

**TABLE 1:** Core journals ranked by number of records.

**Source titles**	**Records**	**% of 5561**
Journal of American College	270	4.86
Psychological Reports	119	2.14
Journal of College Student Development	102	1.83
Journal of Counseling Psychology	83	1.49
Addictive Behaviors	60	1.08
Personality and Individual Differences	48	0.86
Social Behavior and Personality	45	0.81
Plos One	44	0.79
Journal of Affective Disorders	41	0.74
Journal of Clinical Psychology	40	0.72

The top research areas contributing to the publication of research on the mental health and well-being of university students are presented in Table 2. Nearly half of the records in the dataset are published in *psychology* journals. Another influential research area in the field is *psychiatry*, which captures almost 20% of the publications. Journals on *education and educational research* also accumulate a considerable number of publications in the field (15%). Other relevant research areas in the field are connected with health and medicine, including *public environmental occupational health*, *substance abuse*, *general internal medicine*, *neurosciences neurology*, *health care sciences services*, and *nursing*. Finally, the field is also grounded, although to a lower extent, in the publications emerging from journals in the *social sciences*, *family studies*, and *social work* research.

**TABLE 2:** Top research areas ranked by number of records.

**Research areas**	**Records**	**% of 5561**
Psychology	2,456	44.2
Psychiatry	1,017	18.3
Education Educational research	857	15.4
Public environmental occupational health	768	13.8
Substance abuse	268	4.8
Social sciences other topics	223	4.0
General internal medicine	203	3.6
Neurosciences neurology	167	3.0
Health care sciences services	160	2.9
Nursing	152	2.7
Family studies	128	2.3
Social work	116	2.0

All in all, the productivity analysis for journals and research areas showed that most research on mental health and well-being in university students is disseminated in journals in the “psy disciplines”’ (i.e., psychology and psychiatry) (McAvoy, 2014), which is consistent with previous research on mental health in general populations (e.g., Haslam and Lusher, 2011). However, our findings demonstrated that the volume of research in psychology doubles that of research emerging from psychiatric journals. This contrasts with the findings by Haslam and Lusher (2011), who demonstrated that psychiatry journals had a greater influence on mental health research compared to clinical psychology journals and that psychiatry journals accumulate a higher volume of research and citations on mental health research. This is probably because our study includes publications emerging from all branches of psychology, unlike the study by Haslam and Lusher, which included only journals in the field of clinical psychology. Additionally, mental health services in higher education are typically provided by counseling centers led and staffed by non-medical professionals (e.g., psychologists, social workers, counselors, and family therapists) who tend to adopt developmental models of practice grounded in the behavioral sciences and focused on adjustment issues, vocational training, employment, and other personal needs rather than diagnosis and symptom reduction, more common in the biomedical sciences (i.e., psychiatry) (LeViness et al., 2018; Mitchell et al., 2019).

### Productivity II: Leading Authors and Countries/Territories

The 5,561 publications in the dataset were published by a total of 16,161 authors from 119 countries worldwide. Table 3 shows the researchers with the highest number of publications in the field. D. Eisenberg appears as the most productive researcher, followed by K. Peltzer and S. Pengpid. Authors on the list come from diverse geographical backgrounds. Five of the authors work at three different American universities (University of Michigan, Harvard Medical School, and Boston University), two researchers work at KU Leuven University (Belgium), and two other authors are affiliated to the same two universities in Thailand and South Africa. Other prolific researchers are affiliated with higher education institutions in the Netherlands, Egypt, and Germany.

**TABLE 3:** Leading authors ranked by number of records.

**Authors**	**Organization**	**Country/territory**	**Records**
Eisenberg, D	University of Michigan	United States	36
Peltzer, K	Mahidol University;	Thailand;	35
	University of Limpopo	South Africa	
Pengpid, S	Mahidol University;	Thailand;	33
	University of Limpopo	South Africa	
Brauffaerts, R	KU Leuven University	Belgium	30
Auerbach, RP	Harvard Medical School	United States	29
Kessler, RC	Harvard Medical School	United States	28
Cuijpers, P	Vrije University Amsterdam	Netherlands	25
Mortier, P	KU Leuven University	Belgium	23
Ebert, DD	Friedrich-Alexander University of Erlangen-Nürnberg	Germany	22
Green, JG	Boston University	United States	22
Abdel-Khalek, AM	University of Alexandria	Egypt	21
Chang, EC	University of Michigan	United States	21

Countries and territories leading research on mental health and well-being of university students are presented in Table 4. The United States is the indisputable leader in this field, publishing more than half of the records in the dataset. This is nearly 10 times the number of publications produced in China, which occupies the second position in the ranking and accounts for nearly 6% of the volume of research in the dataset. Three predominantly English speaking countries/territories complete the top five of the ranking: Canada (265 records), Australia (254), and England (243). The rest of the countries in the list are situated in Europe (Spain, Germany, Turkey), Western Asia (Iran), Africa (South Africa), and East Asia (Japan), which demonstrates that research on college students’ mental health and well-being is a matter of concern in different regions of the world, at least to some extent.

**TABLE 4:** Leading countries/territories ranked by number of records.

**Countries/territories**	**Records**	**% of 5561**
United States	2934	52.8
Peoples’ Republic of China	329	5.9
Canada	265	4.8
Australia	254	4.6
England	243	4.4
Turkey	218	3.9
Spain	190	3.4
Iran	126	2.3
South Africa	122	2.3
Germany	120	2.2
Japan	117	2.1

Overall, the productivity analysis for authors and countries indicated that the research of mental health and well-being of university students occurs in a variety of locations around the world, especially in developed countries, and in a very prominent way, in the United States. This is not surprising since it is in those countries where better infrastructures and more abundant resources for research are available (Wong et al., 2006), and a more lasting tradition in the study of mental health, in general, exists (Gopalkrishnan, 2018). However, Larivière et al. (2013) found that the productivity of the United States on mental health research has dropped significantly and remained stable in other two English speaking countries (the United Kingdom and Canada) since 1980. On the contrary, the number of publications from European countries and the five major emerging national economies (Brazil, Russia, India, China, and South Africa), has experienced remarkable growth, and collectively account nearly for half of the publications in the field. Still, the predominance of knowledge generated in the developed world today, which tends to be grounded on psychiatric and psychological perspectives, might be eclipsing non-traditional views on mental health and well-being that are popular in other regions of the world and, therefore, limiting the development of effective initiatives that align better with local norms, values, and needs in LMICs (Timimi, 2010; Summerfield, 2013).

### Social Structure: Networks of Scientific Collaboration

Research collaboration is regarded as an indicator of quality research and a means to improve research productivity and academic impact (i.e., citations) (Kim, 2006; Abramo et al., 2009). In particular, international research collaboration is considered a key contributor to the social construction of science and the evolution of scientific disciplines (Coccia and Wang, 2016). There is recent evidence that national and international research collaborations have been accelerating in recent years (Gazni et al., 2012; Wagner et al., 2015), especially in applied fields such as medical and psychological disciplines (Coccia and Bozeman, 2016). In this study, co-authorship analyses were performed to find out patterns in the scientific collaboration between researchers and countries/territories on the mental health and well-being of university students.

**FIGURE 3:**
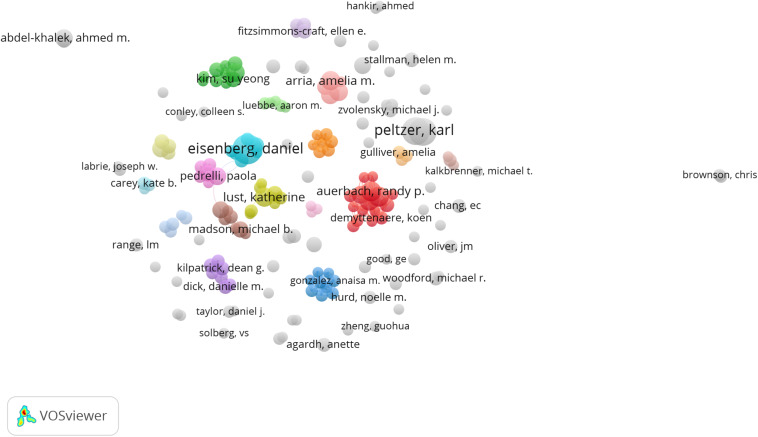
Collaborative research networks between researchers. Only researchers with five or more publications were considered in the analysis (*n* = 179).

Figure 3 demonstrates collaborative ties among authors who published at least 5 articles in the dataset (*n* = 179). The map shows the existence of multiple productive collaborative networks of five or more researchers contributing to the development of the field. The largest collaboration network (red cluster) represents an international research group composed of 15 scholars affiliated to universities in the United States, Belgium, and Netherlands. This cluster groups some of the leading scholars in the field, including R. P. Auerbach, R. Brauffaerts, R. C. Kressler, and P. Cuijpers. Moreover, researchers in this cluster lead The WHO World Mental Health International College Student (WMH-ICS) Initiative, a large scale international project aimed at promoting the mental health and well-being of college students around the world through generating epidemiological data of mental health issues in university students worldwide, designing web-based interventions for the prevention and promotion of mental health, and disseminating evidence-based interventions (Cuijpers et al., 2019). The second biggest cluster (green) represents an intra-national research network that includes 10 researchers from eight different higher education institutions in the United States. The dark blue cluster represents an institutional collaborative network, including nine researchers from the School of Public Health, Puerto Rico. Other prominent clusters in the map represent collaborative research networks between eight (olive color) and seven researchers (turquoise, violet, orange, and mellow mauve). This contrasts, however, with the limited collaboration that exists between clusters. Only four of the clusters on the map demonstrate some kind of scientific collaboration in the field (light blue, pink, brown, and yellow).

**FIGURE 4:**
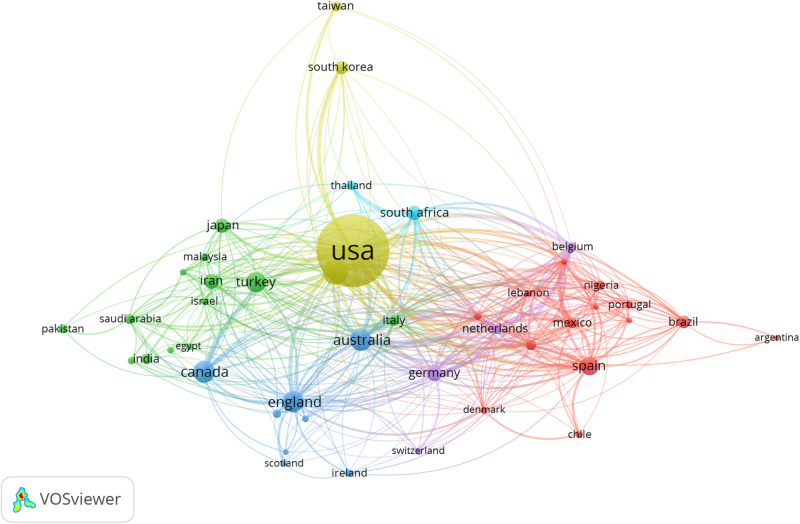
Collaborative research networks between countries and territories. Only countries with 20 or more publications were considered in the analysis (*n* = 45).

Cross-country collaboration networks in mental health and well-being of university students study are presented in Figure 4. Research collaborations between countries with 20 or more publications were considered in this analysis (*n* = 45). The United States occupies the central position of the map and shares collaborative ties with all other countries/territories, forming a cluster together with China, South Korea, and Taiwan. Overall, the results suggest that international collaborations in the field are framed to a large extent by cultural, linguistic, and geographical proximity. For instance, the largest cluster (red) is formed by two European countries (Spain and Portugal) and other South American countries with whom they share historical and cultural backgrounds. Other European countries form the purple cluster. Similarly, the blue cluster clearly brings together predominantly English-speaking countries and territories, while the green cluster agglomerates a range of Asian countries.

Collectively, the results of our study suggest that research collaboration in the field of mental health and well-being in university students remains relatively scarce and localized to date. The social structure of the field at the author level could be described as an archipelago formed by a large number of islands (research groups) of different composition and size but with few bridges connecting them, which suggests a relatively fragmented research community. Moreover, while the existence of international collaborative networks was evident in the analysis, they seem to be formed within national borders, between researchers in neighboring countries/territories, or between countries that share cultural, linguistic, and historical heritages. This may be due to the important role that cultural and traditional values play in the conceptualization of mental health and well-being across contexts (Eshun and Gurung, 2009; Vaillant, 2012; Fernando, 2019). Also, language differences, divergent cross-national institutional and organizational traditions, and increased costs of extramural collaboration, have been found to complicate the formation and continuity of research partnerships in health research (Hooper et al., 2005; Freshwater et al., 2006). Nevertheless, limited within- and between-country research collaboration arguably poses challenges to the development of a field in terms of lost opportunities to challenge assumptions taken for granted and move toward fresh perspectives, push boundaries in methods and techniques, meet diverse groups of people from differing cultures and get immersed in those cultures, share information, resources, and skills, and address common mental health problems through the pooling of resources (Rolfe et al., 2004; Freshwater et al., 2006).

### Intellectual Structure: Disciplines Underlying the Foundations of the Field

Interdisciplinarity is considered as a valuable approach to address the complex and multidimensional nature of health and well-being (Mabry et al., 2008). Buckton (2015) argues that the integration of medical, psychological, and social sciences have contributed to generate “new insights into theory, practice, and research in mental health and development.” (3). To examine the disciplines underlying research on the mental health and well-being of university students, a journal co-citation analysis was performed. In this analysis, only journals with at least 50 citations were considered (*n* = 593). The nodes on the map represent journals and their size reflects the number of co-citation relationships with other journals. Colors account for journal clusters, which agglutinate journals with higher co-citation relationships and stronger semantic connectedness. Clusters were interpreted and labeled accounting for the WoS categorization of the journals with the highest co-citation links within each cluster. For example, if the *Journal of Personality and Social Psychology*, the *Journal of Counseling Psychology*, and *Personality and Individual Differences* clustered together, this group was interpreted as the personality, social, and counseling psychology cluster.

**FIGURE 5:**
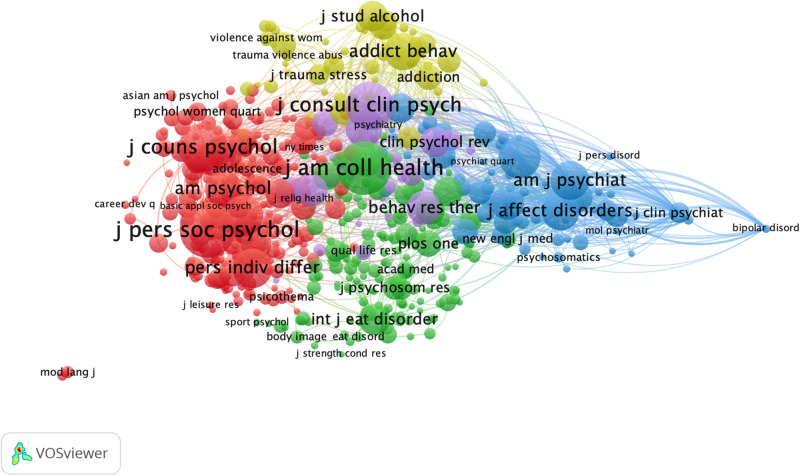
Map of clustered network journals based on co-citation data. Only publications with 50 or more citations were considered in the analysis (*n* = 593).

In general, the findings of this study suggest that research on mental health and well-being in university students is interdisciplinary, to a certain extent, and mainly emerges from the convergence of research conducted in the behavioral and biomedical sciences, as it has been suggested elsewhere (Schumann et al., 2014; Wittchen et al., 2014b). More specifically, the map shows that the research in the mental health and well-being of university students is constructed through the integration of knowledge generated in five interconnected disciplines (see Figure 5). To the left of the map, the red cluster integrates journals on *personal, social, and counseling psychology*. To the right, the blue cluster represents the contribution of *psychiatric journals* to research to the formation and development of the field. At the top, the yellow cluster groups journals on *substance abuse* and issues related to alcohol consumption, addiction, and interpersonal violence. At the bottom of the map, journals covering topics on eating behaviors, sleep, and other issues related to *physical health* converge on the green cluster. At the center of the map is the purple cluster, which includes journals in the area of *clinical psychology and behavioral therapy*.

More broadly, the findings suggest that biomedical sciences contribute to a large extent to the composition of the field. Psychiatric research emerged in our study as an obvious building block in the study of university students’ mental health and well-being, which is not surprising considering the historical contributions of biomedical disciplines to mental health research (Schwartz and Corcoran, 2010). Within the behavioral sciences, personality and social psychology, which explores processes and mechanisms through which social phenomena influence mental health and well-being (Sánchez Moreno and Barrón López de Roda, 2003), appears as a key discipline underlying the foundations of the field. Surprisingly, clinical psychology journals occupy a central position in the map and demonstrate co-citation relationships with journals from all other clusters but make up the most dispersed network and account for a considerably lower volume of co-citation relationships in the field. This suggests that clinical psychology journals are more subordinate to journals in other disciplines in terms of citations flows, and ultimately, play a less unique role in research on the mental health and well-being of university students, as suggested by Haslam and Lusher (2011). Interestingly, research arising from the social sciences (e.g., sociology and anthropology) does not seem to make a distinctive contribution to the intellectual structure of the field, which suggests that the influence of social contexts and cultures on university students’ mental health and well-being (e.g., inequality, social norms, public policies, cultural beliefs, and values) is an underexplored research area. Still, the density of co-citation network relationships within and between clusters is particularly noteworthy, considering the lack of common language between disciplines, the absence of a shared philosophy of practice on mental health, and the tensions between medical, psychological, and social explanations of mental distress (Bailey, 2012).

### Conceptual Structure: Topical Foci Addressed in the Literature Over the Last 45 Years

**FIGURE 6:**
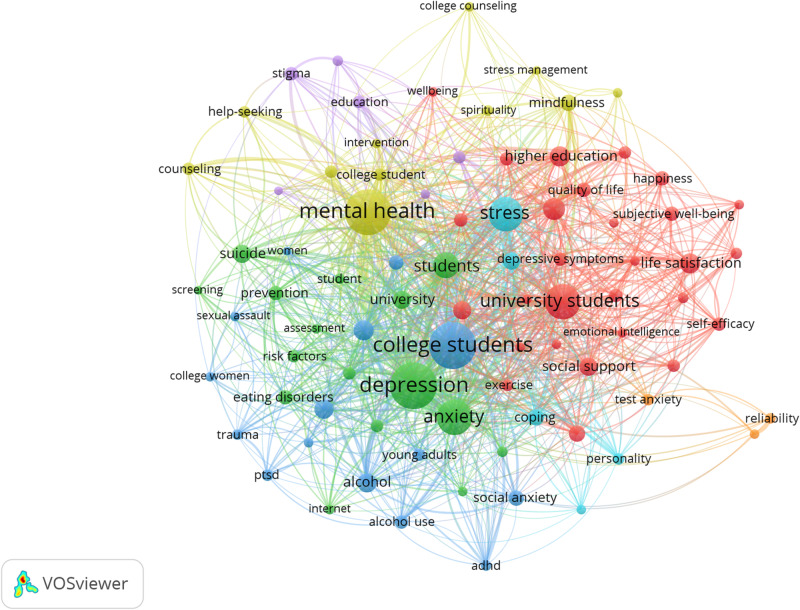
Topical foci in mental health and well-being of university students research. Only keywords with 25 or more occurrences were considered in the analysis (*n* = 84).

The topical foci of research on the mental health and well-being of university students during the 1975–January 2020 period are presented in Figure 6. The map offers a visual representation of the co-occurrence analysis of author keywords of all the publications included in the dataset. Only the most frequently occurring keywords (25+ occurrences) were considered in the analysis (*n* = 84). Items that were not related to others and do not belong to the existing clusters were excluded. The size of the nodes indicates the occurrence of author keywords in the dataset and the thickness of edges represents the co-occurrence strength between pairs of keywords.

The most frequent keywords in the dataset, excluding students’ descriptors (e.g., college students and university students), refer to common mental health challenges experienced by university students such as depression (*n* = 612), anxiety (*n* = 353), and stress (*n* = 341). Salutogenic-related keywords such as well-being and life satisfaction occurred less often (*n* = 138, *n* = 113, respectively), suggesting that pathogenic approaches to the exploration of mental health issues in higher education are more widespread. More broadly, seven general themes seem to summarize the topical foci of interest in the field of mental health and well-being of university students over the last 45 years. First, there has been a general interest in *positive mental health*, as denoted by frequently co-occurring key terms such as well-being, self-esteem, life satisfaction, social support, emotional intelligence, and happiness (red cluster). Second, *mental disorders* stand as another theme widely addressed in the literature, with a special emphasis on depression, anxiety, and to a lesser extent, suicide and suicidal ideation (green cluster). A third topical area in this field has been *substance abuse*, most predominantly alcohol consumption (blue cluster). The fourth theme reflects college *counseling for mental health*, including interventions and protective factors such as mindfulness, stress management, spirituality, and help-seeking (yellow cluster). Other topics reflected in the map are *mental illness stigma* (purple), *stress* (e.g., psychological distress and coping) (light blue), and *mental health measurement* (orange).

## Conclusion

This study provides a comprehensive overview of the research on university students’ mental health and well-being in the last 45 years using bibliometric indicators. In general, the results reveal interesting trends in the evolution of the field over the last four decades and promising scientific patterns toward a better understanding of the mental health and well-being of university students internationally. First, the interest in the mental health and well-being of university students has grown in the last decades and in a very significant way during the last 10 years, indicating that this area has not still reached its maturity period and will continue developing in the future. Second, research in the field is relatively interdisciplinary and emerges from the convergence of research conducted in several disciplines within the behavioral and biomedical sciences. Third, research in this field is produced by a community of productive researchers coming from several regions around the world, most notably in the United States, which secures a generation of scholars that will continue shaping the field in the years to come. Fourth, over the last 45 years, researchers have been able to address a multitude of research topics in the field, including positive mental health, mental disorders, substance abuse, counseling, stigma, stress, and mental health measurement.

However, this study also identified some issues that could be hindering the development of the study of the mental health and well-being of university students. For example, the research available overrepresents theoretical and disciplinary approaches from the developed world. Additional studies on the field from developing economies and LMICs are needed to provide a more comprehensive picture and ensure a fair representation of the multiple perspectives available in the field. Such studies would inform administrators and practitioners on how to broaden and enrich available programs and initiatives to promote mental health and well-being in higher education contexts in order to offer alternative forms of support that university students find appropriate for their social and cultural values. Moreover, the research community contributing to the development of the field is relatively fragmented. There are multiple research groups but little research collaborations between them and, at the international level, these connections tend to be limited by geographic, cultural, and language proximity. In this context, more actions like the WMH-ICS Initiative could provide a partial solution to this problem by strengthening national and international research partnerships and facilitating knowledge exchange across regions. Also, special issues in the core journals in the field inviting cross-cultural studies on the topic could contribute to promoting research collaboration across regions and research in less represented countries. The field would also benefit from a greater volume of research from the social sciences and humanities exploring the influence of social, cultural, economic, and educational factors on the conceptualization, manifestation, and experience of mental health and well-being. Moreover, more studies emerging from disciplines such as sociology, anthropology, business, and education, would likely increase the permeability of positive mental health concepts into the field and contribute to the promotion of salutogenic approaches to the study of mental health and well-being of university students.

This study has several limitations. First, publications were retrieved only from the WoS database, which limits the generalizability of the findings. Second, WoS provides stronger coverage of Life Sciences, Biomedical Sciences, and Engineering, and includes a disproportionate number of publications in the English language (Mongeon and Paul-Hus, 2016). This could partially explain the low number of publications emerging from the Social Sciences, the Arts, and the Humanities, and research conducted in non-English speaking countries in the present study. Third, only journal articles were retrieved for analysis, excluding other relevant publications in the field such as reviews, book chapters, and conference proceedings. Future studies could replicate the findings of this study using alternative databases (e.g., Scopus and PubMed) or a combination of them, as well as different filters in the search strategy, to provide an alternative coverage of research conducted in the field. Nevertheless, we believe that the bibliometric approach used in this study offers novel insights about the development and current status of the field and some of the challenges that undermine its progression.

## Data Availability Statement

The datasets generated for this study are available on request to the corresponding author.

## Author Contributions

DH-T and LI contributed to conception and design of the study, organized the database, and performed the statistical analysis. DH-T, LI, and JS wrote the first draft of the manuscript. NL, AC, AA, YN, and AM wrote the sections of the manuscript.

## Conflict of Interest

The authors declare that the research was conducted in the absence of any commercial or financial relationships that could be construed as a potential conflict of interest.

## Footnotes

1 www.vosviewer.com
